# Hidden blood loss and the influential factors after minimally invasive treatment of posterior pelvic ring injury with sacroiliac screw

**DOI:** 10.1186/s13063-024-08147-0

**Published:** 2024-05-06

**Authors:** Ding Xu, Min Liu, Weigang Lou, Ming Li, Jingwei Xiao, Hongbao Wu, Yunqiang Zhuang, Jianming Chen

**Affiliations:** 1 Department of Orthopedic Trauma Surgery, Ningbo NO.6 Hospital, 1059 East Zhongshan Road, Ningbo, China; 2 Department of Orthopedics, Third Affiliated Hospital of Wenzhou Medical University, Wenzhou, China; 3 Ningbo University Medical College, Ningbo, 315211 China

**Keywords:** HBL, Posterior pelvic ring injury, Sacroiliac screw, Influential factors

## Abstract

**Background:**

To analyze the perioperative bleeding and hidden blood loss (HBL) of sacroiliac screw minimally invasive treatment of pelvic posterior ring injury and explore the influential factors of HBL after operation for providing reference for clinical treatment.

**Method:**

A retrospective analysis was conducted on data from 369 patients with posterior pelvic ring injuries treated with sacroiliac screws internal fixation at our hospital from January 2015 to January 2022. The research was registered in the Chinese Clinical Trial Registry in July 2022 (ChiCTR2200061866). The total blood loss (TBL) and HBL of patients were counted, and the factors such as gender, age, and surgical duration were statistically analyzed. The influential factors of HBL were analyzed by multiple linear regression.

**Results:**

The TBL was 417.96 ± 98.05 ml, of which the visible blood loss (VBL) was 37.00 ± 9.0 ml and the HBL was 380.96 ± 68.8 ml. The HBL accounted for 91.14 ± 7.36% of the TBL. Gender, surgical duration, fixed position, and fixed depth had significant effects on the HBL (*P* < 0.05).

**Conclusions:**

The HBL was the main cause of anemia after minimally invasive treatment of posterior pelvic ring injury with a sacroiliac screw. Gender, surgical duration, fixed position, and fixed depth were closely related to the occurrence of HBL. In clinical treatment, we should consider these influential factors and take effective measures to reduce the impact of HBL on patients.

**Supplementary Information:**

The online version contains supplementary material available at 10.1186/s13063-024-08147-0.

## Introduction

Pelvic posterior ring injuries were always caused by high-energy violence, and most of them belong to unstable injuries. The treatment had been a headache for clinical orthopedics doctors [1]. The posterior pelvic ring beard 70% of the load of the whole pelvic ring, Posterior pelvic ring injuries such as sacroiliac joint dislocation and sacral fracture were often accompanied by vertical instability and rotational instability [2]. Therefore, early effective reduction and fixation is very necessary. It can restore the stability of the pelvis and significantly reduce the occurrence of complications such as intractable pain and abnormal gait [3–5]. If anatomical reduction and effective fixation cannot be carried out in time, it will lead to poor curative effect and residual dysfunction [6]. Unfortunately, there was no gold standard treatment for the reduction and fixation of the posterior pelvic ring. The specific surgical method, the choice of internal fixation, and the corresponding prognosis were controversial [7]. In 1989, Matta et al. proposed a percutaneous sacroiliac screw (S1 screw) for the treatment of posterior pelvic ring injury, which had been recognized and accepted by more and more clinicians [8]. Biomechanical experiments showed that percutaneous sacroiliac screws had more advantages than posterior sacral rod and anterior plate fixation in shear and rotation resistance, which was in line with the central fixation principle of Biomechanics [9]. At present, percutaneous minimally invasive internal fixation by sacroiliac screw (PMISS) for the treatment of posterior pelvic ring fractures has been widely used in clinic. It has the advantages of minimally invasive, short operation time, and rapid recovery [10]. In the course of disease treatment, we found that patients with posterior pelvic ring injury treated with PMISS often developed anemia. It would affect postoperative functional recovery and increase mortality [11]. Although anemia was closely related to surgical trauma and visible bleeding [12], it was also crucial to pay attention to hidden blood loss (HBL). Because the pelvis had a rich vascular network and a large amount of cancellous bone [13]. HBL was an imperceptible loss of blood. The decrease of hemoglobin (HB) or hematocrit (HCT) in patients was significantly greater than that caused by intraoperative bleeding in theory [14]. Perioperative HBL was very easy to be ignore in clinical work, which had a great impact on the prognosis of patients with pelvic injury, especially elderly patients with basic diseases. After reviewing numerous international journals, there were relatively few studies on HBL related to minimally invasive therapies, with the majority focusing on minimally invasive surgeries of the spine. Research on HBL in minimally invasive treatment of pelvic injuries remained largely unexplored [15–17]. This study retrospectively analyzed the case data of PMISS for the treatment of posterior pelvic ring injury in our hospital from January 2015 to January 2022. By analyzing the perioperative bleeding and HBL of patients to explore the influential factors of HBL after the operation and provide a reference for clinical prevention and treatment.

## Patients and methods

### Patients

**Fig. 1:**
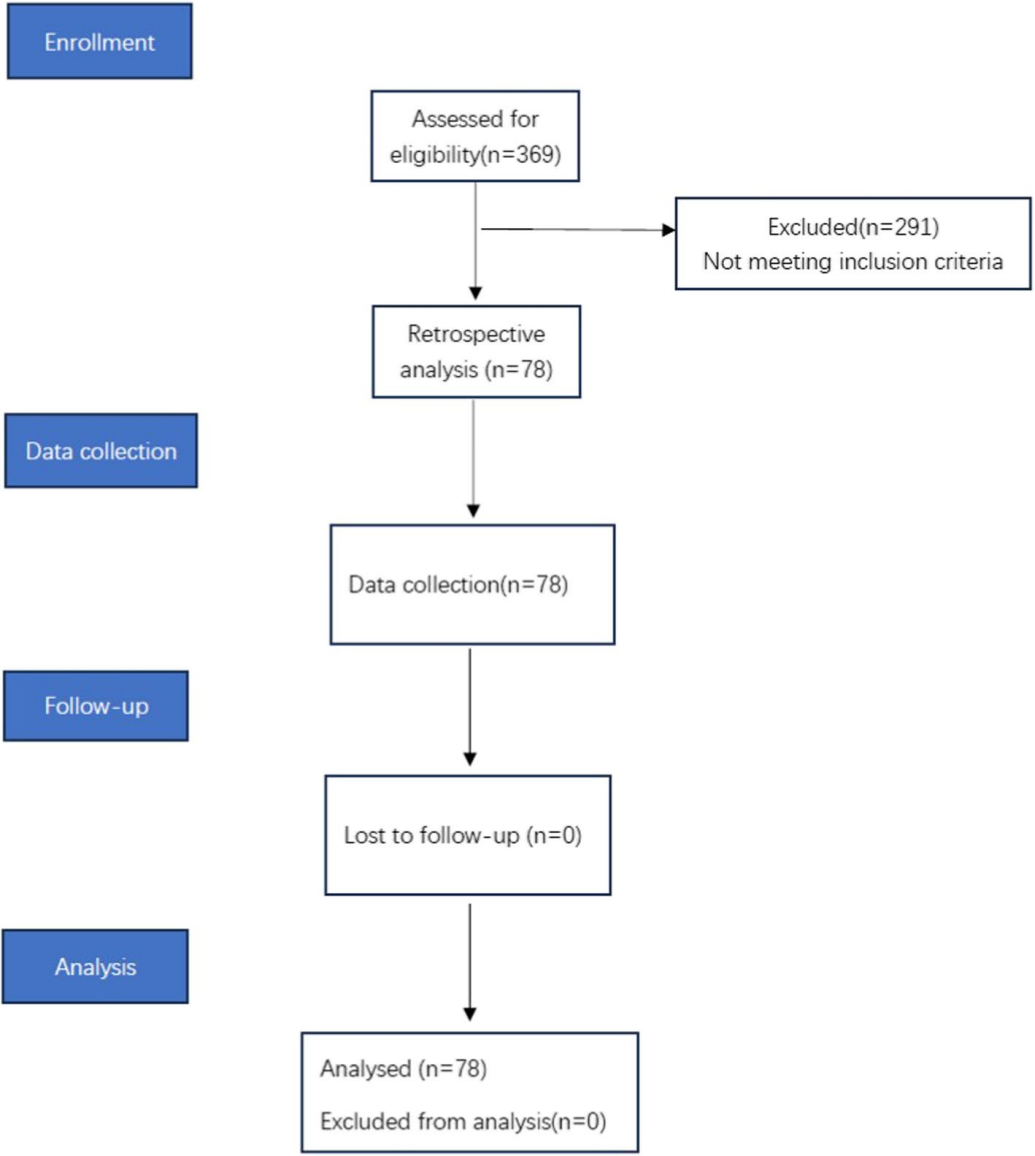
CONSORT flow chart for patient recruitment

A retrospective analysis was conducted on data from 369 patients with posterior pelvic ring injuries treated with PMISS at our hospital from January 2015 to January 2022. This sample size was calculated using Solvin’s formula. Inclusion criteria are as follows: (1) patients with posterior pelvic ring injury and sacroiliac screw fixation; (2) AO/OTA classification: 61b, 61c1.2, 61C1. 3 [18]; (3) blood examination was performed before and after operation; (4) no history of thrombosis, abnormal coagulation function, and other high-risk hemorrhagic diseases; (5) the clinical data were complete; (6) signed informed consent. Exclusion criteria are as follows: (1) accompanied by anterior fixation (except staged operation with external fixator); (2) old fracture and pathological fracture; (3) AO/OTA classification: 61A, 61c1. 1, 61C2, 61C3; (4) history of preoperative blood transfusion and long-term use of anticoagulants; (5) chronic hemorrhagic diseases, hematological diseases, and malignant tumors; (6) incomplete clinical data; (7) the rehydration volume was more than 2000 m1 [19] within 24 h after operation. According to the exclusion criteria, a total of 291 patients were excluded from the study. Among them, 276 patients underwent plate fixation of the anterior pelvic ring, 7 cases were rehydrated more than 2000 ml within 24 h after operation, 4 cases had incomplete clinical data, and 4 cases received a preoperative blood transfusion. The CONSORT flow chart of the included patients is shown in Fig. 1. Therefore, a total of 78 patients were included in this study with an average of (47.35 ± 5.23) years. There were 42 males and 36 females. The study protocol was approved by Ningbo NO.6 Hospital Institutional Review Board (No. L2022090). The CONSORT checklist of this study protocol are shown in Supplementary Material 1. All methods were performed in accordance with the relevant guidelines and regulations. All patients and their families signed the informed consent.

### Surgical method

All patients needed a clean enema the night before the operation. Under general anesthesia, the patient took the flat position and underwent C-arm fluoroscopy positioning (pelvic entrance position, exit position, and lateral position). An incision was made about 1 cm on the side of the posterior superior iliac spine, separated the soft tissue to the iliac bone, and then placed the positioning sleeve. The guide needle with a diameter of 2 mm was vertically inserted into the posterior side of the iliac wing. and the S1 vertebral body was inserted between the superior sacral notch and the S1 anterior fissure through the sacroiliac joint space, the sacral auricular surface, and the sacral wing. Through the sacroiliac joint space, sacral auricular surface, and sacral wing, the guide needle entered the S1 vertebral body. During the placement of the guide needle, we need to keep the guide needle parallel to the superior sacral notch and tilt from the rear outside to the front inside at an angle (25°) to the coronal plane of the pelvis. The C-arm fluoroscopy should be repeated to observe and adjust the position of the guide needle in the process of needle threading. According to the need of fixation, another screw can be fixed in the S1 vertebral body repeatedly or screwed at the S2 level. All hollow lag screws were 6.5 mm in diameter and were produced by China company (Weigao).

### Postoperative treatment

Antibiotics were applied twice within 24 h after operation. Recheck blood routine and give blood transfusion according to anemia. Lower limb activities began on the second day after the operation. After lying in bed for 4–6 weeks, the patient started to move out of bed and gradually walk with weight on the affected limb. The X-ray would be checked regularly.

### Data collection

**Fig. 2:**
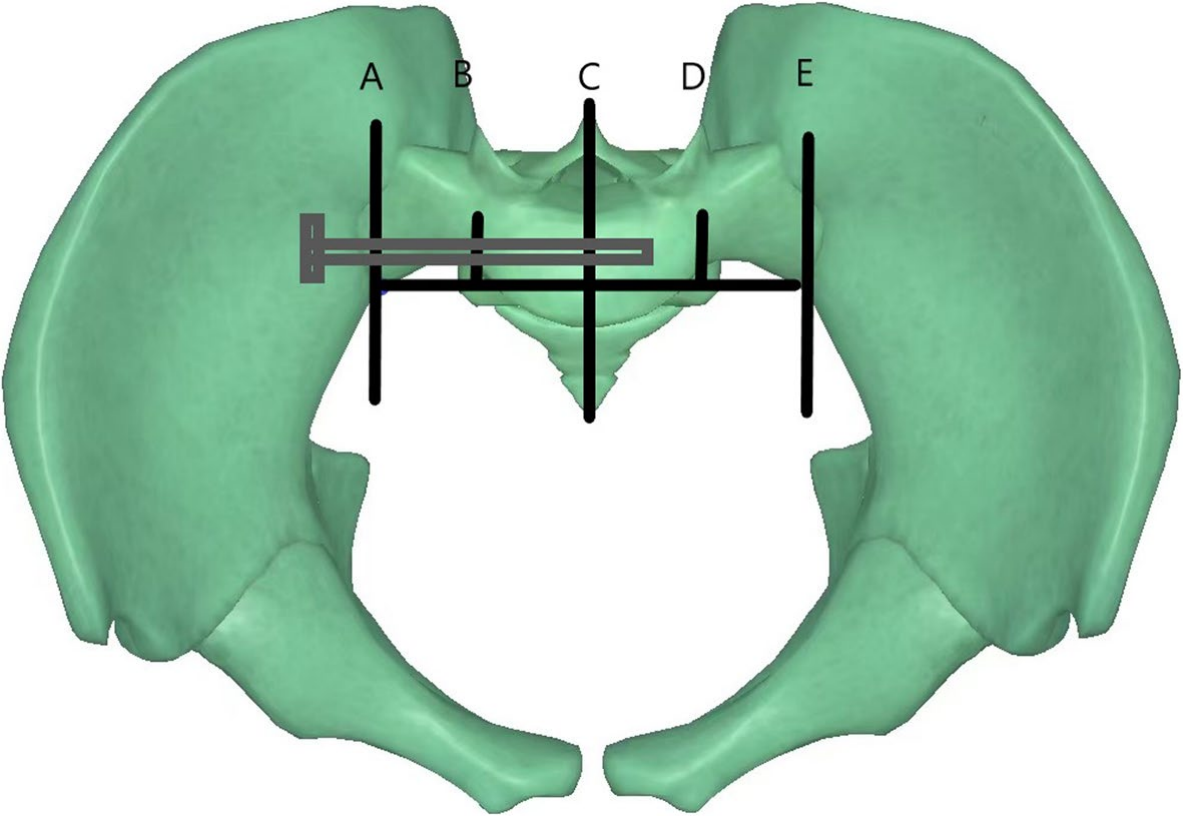
Definition of screw depth: make a connecting line at the front edge of the sacroiliac joint on both sides at the pelvic entrance position and divide it into four equal parts. The sacroiliac screw head can reach area AB, area BC, area CD and area DE, which are I-degree, II-degree, III-degree, and IV-degree, respectively

The basic information of patients from the electronic medical record system of our hospital was collected, including the patient’s age, gender, body mass index (BMI), surgical duration, time to surgery, anesthesia, anemia, blood transfusion history, smoking history, alcohol, hypertension, diabetes, cardiovascular and cerebrovascular diseases, HB, HCT, albumin, type of posterior pelvic ring injury, fixed position, and depth (Fig. 2). The injury of the posterior pelvic ring was divided into three types. Type I was an unstable fracture of the iliac wing which can be combined with partial injury of the sacroiliac joint. Type II was an unstable injury of the sacroiliac joint and type III was an unstable fracture of the sacrum. All indicators were measured by the same researcher 3 times, and then the average value was taken to reduce the error. Hemoglobin was used to assess the patient’s anemia. According to the definition of the World Health Organization, men < 130 g/L and women < 120 g/L were defined as anemia [20].

### Blood volume calculation

The blood volume (BV) of the patient was calculated according to the equation proposed by Nadler et al. [21]:

$${\text{BV}}={\text{k}}1\times {\text{H}}3+{\text{k}}2\times {\text{W}}+{\text{k}}3$$

(BV: the preoperative blood volume (L); H: height (m); W: body mass (kg); male: k1 = 0.3669, k2 = 0.03219, K3 = 0.6041; female: k1 = 0.3561, k2 = 0.03308, k3 = 0.1833).

### Visible blood loss (VBL)

VBL = blood volume in the suction bottle + increased blood volume such as gauze − flushing fluid volume.

### Total blood loss (TBL)

The loss of red blood cells in postoperative blood can be calculated by the difference between preoperative and postoperative HCT.

Red blood cell loss = BV × (HCT _before_ and HCT _after_) + blood transfusion.

TBL = red blood cell loss (L)/HCT mean [22].

(HCT mean = 1/2 × (HCT _before_ + HCT _after_), the results of the HCT examination on the morning of the second day after admission were HCT _before_, and the results of the HCT examination on the morning of the third day were HCT_after_).

### Hidden blood loss (HBL)

HBL = TBL − VBL.

### Hidden blood loss ratio

HBL ratio = HBL/TBL.

### Statistical analysis

All data were described and analyzed by SPSS 22.0 (IBM, USA). The normality test of measurement data was carried out by Kolmogorov Smirnov. The description of measurement data adopted xˉ ± s or median (interquartile range) according to the data distribution type. The Pearson correlation test was used to analyze measurement data. Rate or percentage was used to describe counting data. At the same time, the Spearman correlation was used for statistical analysis. The variables with high correlation were selected, and the independent risk factors related to hidden blood loss were determined by hierarchical regression analysis. A positive coefficient indicated a positive effect on the dependent variable (hidden blood loss), while a negative coefficient indicated a negative effect on the dependent variable. *P* < 0.05 was considered statistically significant.

## Results

### Perioperative bleeding volume

In this study, the TBL of patients was 417.96 ± 98.05 ml, of which the VBL was 37.00 ± 9.0 ml and the HBL was 380.96 ± 68.8 ml. The HBL accounted for 91.14 ± 7.36% of the TBL (Table 1).

**Table 1:** Perioperative blood loss of patients

Parameters	Mean ± SD
Calculated total blood loss (mL)	417.96 ± 98.05
Visible blood loss (mL)	37.00 ± 9.0
Hidden blood loss (mL)	380.96 ± 68.8
Percentage of hidden loss in total (%)	91.14 ± 7.36

### Influential factors of perioperative HBL

Age, gender, BMI, surgical duration, time to surgery, anesthesia, anemia, blood transfusion history, smoking history, alcohol, hypertension, diabetes, cardiovascular and cerebrovascular diseases, pulmonary diseases, perioperative gastrointestinal bleeding, chronic anticoagulation, HB, HCT, albumin, type of posterior pelvic ring injury, fixed position, and depth were included in the analysis of possible influential factors of HBL in our study (Table 2). All patients were operated on under general anesthesia, and all patients had anemia after the operation, so the factors of anesthesia and postoperative anemia were excluded. Pearson correlation test analyzed age, BMI, surgical duration, time to surgery, HB, HCT, and albumin. BMI and surgical duration were significantly correlated with HBL (*P* < 0.05) (Table 3). Spearman correlation test statistically analyzed gender, anemia (preoperative), blood transfusion history, smoking history, alcohol, hypertension, diabetes, cardiovascular and cerebrovascular diseases, pulmonary diseases, perioperative gastrointestinal bleeding, chronic anticoagulation, type of posterior pelvic ring injury, fixed position and depth. We found the gender, fixed position, and fixed depth had significant effects on the HBL (*P* < 0.05) (Table 4). Finally, BMI, surgical duration, gender, fixed position, and fixed depth were included in multiple linear regression analysis. It was found that surgical duration, gender, fixed position, and fixed depth had significant effects on HBL (*P* < 0.05) (Table 5).

**Table 2:** Patient’s demographic information

Parameters	Value	Percentage/range
Median age (year)	47.35 ± 5.23	23.00–72.00
Gender (male/female) (*n*)	42/36	53.84%/46.16%
BMI (kg/m^2^)	23.34 ± 2.12	20.03–26.78
Smoking history (yes/no)	18/60	23.07%/76.93%
Alcohol (yes/no)	20/58	25.64%/74.36%
Surgical duration (min)	65.00 ± 14.56	45.00–120.00
Time to surgery (days)	3.50 ± 1.50	2.00–8.00
Anesthesia (general)	78	100%
Anemia		
Preoperative	56	71.79%
Postoperative	78	100%
Transfusion	9	11.53%
Comorbid conditions		
Hypertension	14	17.94%
Diabetes mellitus	17	21.79%
Cerebrovascular disease	9	11.53%
Cardiovascular disease	13	16.66%
Pulmonary disease	7	8.97%
Perioperative gastrointestinal bleeding/ulcer	1	1.28%
Chronic anticoagulation preoperatively	11	14.10%
Preoperative hematocrit (%)	34.50 ± 0.29	26.70–42.30
Preoperative hemoglobin (g/L)	113.00 ± 5.38	73.00–145.00
Preoperative albumin (g/L)	37.91 ± 3.16	25.20–46.60
Type of posterior pelvic ring injury		
Type I	14	17.94%
Type II	39	50.00%
Type III	25	32.06%
Fixed position		
S1	65	83.3%
S2	6	7.69%
S1 and S2	7	8.97%
Fixed depth		
I	0	
II	34	43.58%
III	37	47.43%
IV	7	8.97%

**Table 3:** Results of Pearson correlation analysis for hidden blood loss

Parameters	Pearson’s r	*P*-value
Median age (year)	− 0.403	0.371
BMI (kg/m^2^)	0.820	**0.024**
Surgical duration (min)	0.024	**0.034**
Time to surgery (days)	− 0.177	0.774
Preoperative hematocrit	0.449	0.312
Preoperative hemoglobin (g/L)	0.442	0.321
Preoperative albumin (g/L)	0.227	0.547

*Abbreviation: BMI* body mass index

**Table 4:** Results of Spearman correlation analysis for hidden blood loss

Parameters	Sig, (2-tailed)	*P*-value
Gender	− 0.285	**0.011**
Smoking history	− 0.048	0.676
Alcohol	0.177	0.120
Anemia		
Preoperative	− 0.130	0.255
Transfusion	0.077	0.504
Comorbid conditions		
Hypertension	− 0.022	0.852
Diabetes mellitus	0.040	0.728
Cerebrovascular disease	0.169	0.140
Cardiovascular disease	0.088	0.444
Pulmonary disease	− 0.050	0.667
Perioperative gastrointestinal bleeding/ulcer	0.175	0.126
Chronic anticoagulation preoperatively	0.025	0.825
Type of posterior pelvic ring injury	− 0.028	0.805
Fixed position	0.282	**0.012**
Fixed depth	0.345	**0.002**

**Table 5:** Results of hierarchical regression analysis for hidden blood loss

	Model 1			Model 2		
	*β*	*t*	Sig	*β*	*t*	Sig
BMI (kg/m^2^)	− 0.245	− 2.200	0.051	− 0.104	− 1.097	0.276
Surgical duration (min)				0.272	2.952	**0.004**
Gender				− 0.273	− 2.957	**0.004**
Fixed position				0.329	3.398	**0.001**
Fixed depth				0.266	2.847	**0.006**
*R*^2^	0.060			0.397		
△*R*^2^	0.060			0.337		
*F*	4.842			9.471		

*Abbreviation: BMI* body mass index

## Discussion

The posterior pelvic ring was also known as the sacroiliac complex. It was a stable structure formed by the sacrum and bilateral iliac bones through the interosseous sacroiliac ligament, anterior and posterior sacroiliac ligament, sacral tubercle ligament, sacral spine ligament, and iliolumbar ligament, which played a vital role in maintaining the stability of the whole pelvis [23]. Arterial anastomosis widely existed in the pelvis, and the inner wall of the pelvis was covered with venous plexus. The arteries and veins were connected with each other and there was no venous valve. Once damaged, it was easy to cause extensive bleeding. Our study also found that 71.79% of patients with posterior pelvic ring injury had preoperative anemia, while the incidence of postoperative anemia after PMISS was 100%. This was a cognitive gap with our usual view that PMISS of posterior pelvic ring injury had less bleeding and rapid recovery. We tended to focus on VBL, which was easy to ignore for hidden bleeding. The HBL was first observed by Pattison et al. after knee arthroplasty. Subsequently, Sehat et al. calculated the real quantitative HBL through a gross equation. They found that HBL accounted for about 50% of the TBL, which was considered to be caused by blood infiltration into tissue space and cell hemolysis [24]. There were many tissue gaps around the pelvis, and the surrounding vascular network was rich. Bleeding was easy to accumulate in the surrounding tissue gaps and causes nonspecific hemolysis to destroy the structure of red blood cells. The destruction of tissue structure led to the loss of function and the reduction of erythrocytes and hemoglobin involved in metabolism. Smorgick et al. found that the percentage of HBL in posterior lumbar surgery was about 39 ~ 42% [25] of the total blood loss, while the percentage of HBL in Xu’s study was higher (47%) [26]. In hip surgery, the percentage of HBL fluctuated from 50 to 80% [27–29]. Compared with the research status of HBL in spine surgery and hip surgery, there were few reports on the injury of the posterior pelvic ring. Our study found that the HBL was 380.96 ± 68.8 ml which accounted for 91.14 ± 7.36% of the TBL (417.96 ± 98.05 ml) in the PMISS of posterior pelvic ring injury. Therefore, HBL may be the main cause of postoperative anemia in patients. PMISS in the treatment of posterior pelvic ring injury has been highly praised in orthopedics because of its advantages of minimally invasive and rapid postoperative recovery [30]. During the whole operation, there was very little bleeding visible to the naked eye which often led to doctors ignoring the attention of patients with postoperative anemia. The results of this study revealed a significantly high probability of postoperative anemia in such patients. It was a significant disparity between this and traditional perceptions of bleeding in minimally invasive pelvic surgeries, with surgeons often assuming minimal blood loss in such procedures. This was also the most meaningful and important finding of this study. Therefore, surgeons needed to enhance their awareness of HBL in minimally invasive pelvic surgeries and take corresponding measures in clinical practice. Surgeons should assess preoperative blood loss, monitor blood routine regularly, and promptly replenish blood volume. Transfusion therapy may be necessary to maintain stable circulating blood volume, reduce complications, and improve the safety and prognosis of surgery. For patients, this can accelerate their recovery. For society, it can save on healthcare expenditures.

This research found that the main influential factors of HBL in PMISS were gender, surgical duration, fixed position, and fixed depth. The HBL was greater in male patients than in female patients, and similar findings were found in many previous studies [31–33]. Male patients seem to be more closely related to perioperative HBL. The probability of requiring a blood transfusion after the operation was also higher than that of women [33]. The reason was not clear. We believed that this was related to the advantages of male patients in body mass index and blood volume. In addition, women tended to reduce hormone secretion and increase osteoclast activity after menopause. This made osteoporosis significantly more severe than in male patients. Osteoporosis could reduce the blood supply to the bone marrow cavity. Therefore, the blood loss during the operation would be reduced too. Surgical duration was also an influential factor of perioperative HBL. The same findings were found in the study of Lei et al. [34, 35]. The extension of operation time meant an increase in the operation step. In the process of sacroiliac screw placement, it was necessary to repeatedly find the most appropriate screw placement direction and depth. Each insertion and extraction of the guide needle would create a nail hole tunnel in the sacroiliac bone, which were potential bleeding points. Moreover, long operation time could also increase the body’s traumatic stress response and cause the release of a large number of inflammatory factors, so as to inhibit the formation of red blood cells and increase the incidence of HBL. The fixed position as the influential factor referred to the simultaneous placement of screws in sacrals 1 and 2, which would prolong the operation time, increase the number of screws and bone channels and naturally increase the corresponding blood loss. The fixed depth was divided into 4 degrees. The different depths could significantly affect the perioperative hidden blood loss. The implantation of the sacroiliac screw first needed the guidance of a guide needle, and then the hollow screw was inserted. The whole process was like the reaming of long bones. The screw head at the front end rotated to expand the bone marrow cavity and moved forward. The deeper the screw, the longer the bone tunnel. At the same time, the hollow design was easy to cause the blood seepage in the bone marrow to flow out into the tissue space through the tunnel after the removal of the guide needle. The longer the tunnel, the more bone marrow is destroyed around it, and the greater the amount of bleeding.

This study was a small sample retrospective study that lacked multi-center and large sample data to provide clinical guidance with more reference value. Pelvic posterior ring injury often involved anterior ring injury and surgery, so it was difficult to collect cases that simply dealt with posterior ring injury. The cases of emergency surgery with anterior ring external fixation were also included ((1) the external fixation had little trauma and the amount of bleeding was almost negligible; (2) external fixation had little interference with our analysis of the HBL in secondary sacroiliac screw fixation of posterior ring injury). Moreover, the research on the pathogenesis was obviously insufficient and needed to be further discussed.

## Conclusions

The blood loss of PMISS in the treatment of posterior pelvic ring injury has received little attention.

This study found that the HBL accounted for more than 90% of the TBL, which was the main cause of anemia in postoperative patients. Gender, surgical duration, fixed position, and fixed depth were closely related to the occurrence of HBL. In clinical treatment, we should consider these influential factors and take positive and effective measures to prevent HBL.

### Supplementary Information

Supplementary Material 1.

## Data Availability

The datasets generated and/or analyzed during the current study are available from the corresponding author on reasonable request.

## Abbreviations

HBL: Hidden blood loss
TBL: Total blood loss
VBL: Visible blood loss
PMISS: Percutaneous minimally invasive internal fixation by sacroiliac screw
HB: Hemoglobin
HCT: Hematocrit
BMI: Body mass index
BV: Blood volume
